# Effects of Valacyclovir on Markers of Disease Progression in Postpartum Women Co-Infected with HIV-1 and Herpes Simplex Virus-2

**DOI:** 10.1371/journal.pone.0038622

**Published:** 2012-06-12

**Authors:** Alison C. Roxby, Alison L. Drake, Francisca Ongecha-Owuor, James N. Kiarie, Barbra Richardson, Daniel N. Matemo, Julie Overbaugh, Sandra Emery, Grace C. John-Stewart, Anna Wald, Carey Farquhar

**Affiliations:** 1 Department of Medicine, University of Washington, Seattle, Washington, United States of America; 2 Department of Global Health, University of Washington, Seattle, Washington, United States of America; 3 Department of Epidemiology, University of Washington, Seattle, Washington, United States of America; 4 Department of Biostatistics, University of Washington, Seattle, Washington, United States of America; 5 Department of Pediatrics, University of Washington, Seattle, Washington, United States of America; 6 Department of Laboratory Medicine, University of Washington, Seattle, Washington, United States of America; 7 Department of Obstetrics and Gynaecology, University of Nairobi, Nairobi, Kenya; 8 Division of Human Biology, Fred Hutchinson Cancer Research Center, Seattle, Washington, United States of America; 9 Division of Vaccine and Infectious Disease, Fred Hutchinson Cancer Research Center, Seattle, Washington, United States of America; 10 Department of Medicine, Therapeutics, and Psychiatry, Kenyatta University, Nairobi, Kenya; UCL Institute of Child Health, University College London, United Kingdom

## Abstract

**Objective:**

Herpes simplex virus type 2 (HSV-2) suppression has been shown to reduce HIV-1 disease progression in non-pregnant women and men, but effects on pregnant and postpartum women have not been described.

**Methods:**

We analyzed data from a cohort of Kenyan women participating in a randomized clinical trial of HSV-2 suppression. Pregnant HIV-1-seropositive, HSV-2-seropositive women who were not eligible for antiretroviral therapy (WHO stage 1–2, CD4>250 cells/µl) were randomized to either 500 mg valacyclovir or placebo twice daily from 34 weeks gestation through 12 months postpartum. Women received zidovudine and single-dose nevirapine for prevention of mother-to-child HIV-1 transmission. HIV-1 progression markers, including CD4 count and plasma HIV-1 RNA levels, were measured serially. Multivariate linear regression was used to compare progression markers between study arms.

**Results:**

Of 148 women randomized, 136 (92%) completed 12 months of postpartum follow-up. While adjusted mean CD4 count at 12 months (565 cells/µl placebo arm, 638 cells/µl valacyclovir arm) increased from antenatal levels in both arms, the mean CD4 count increase was 73 cells/µl higher in the valacyclovir arm than placebo arm (p = 0.03). Mean increase in CD4 count was 154 cells/µl in the valacyclovir arm, almost double the increase of 78 cells/µl in the placebo arm. At 12 months, adjusted HIV-1 RNA levels in the placebo arm increased by 0.66 log_10_ copies/ml from baseline, and increased by only 0.21 log_10_ copies/ml in the valacyclovir arm (0.40 log_10_ copies/ml difference, p = 0.001).

**Conclusion:**

Women randomized to valacyclovir suppressive therapy during pregnancy and postpartum had greater increases in CD4 counts and smaller increases in plasma HIV-1 RNA levels than women in the placebo arm. Valacyclovir suppression during pregnancy and breastfeeding may improve outcomes and delay antiretroviral therapy for HIV-1/HSV-2 co-infected women.

## Introduction

Addressing herpes simplex virus type 2 (HSV-2) co-infection may be a pathway to improving health of HIV-1 infected mothers. More than 80% of HIV-1-seropositive women in sub-Saharan Africa are co-infected with HSV-2 [1]. HSV-2 co-infection has been associated with higher plasma HIV-1 RNA loads, and higher HIV-1 viral set points if HSV-2 infection precedes HIV-1 seroconversion [2], [3]. Trials of herpes virus suppression in HIV-1/HSV-2 co-infected persons have consistently shown reductions in HIV-1 plasma RNA levels, but these trials have excluded pregnant women [4], [5], [6], [7], [8], [9], [10], [11]. Recent randomized clinical trials of acyclovir for herpes suppression in co-infected persons have demonstrated 16–25% reductions in HIV-1 disease progression endpoints over 2 years of follow-up [12], [13]. Progression endpoints for both studies included CD4 thresholds and evidence of clinical disease progression including non-traumatic death and/or ART initiation.

Valacyclovir is now available in generic form, providing higher levels of acyclovir with a simpler regimen, improving adherence and potentially increasing effects on HIV-1 progression. We analyzed data from a randomized, double-blind, placebo-controlled trial to determine whether valacyclovir reduces maternal disease progression, using CD4 count as the primary marker, when taken in late pregnancy and for 12 months postpartum.

## Methods

### Study Design

Participants were part of a randomized, double-blind, placebo-controlled trial of valacyclovir suppressive therapy of 500 mg twice daily in pregnant HIV-1/HSV-2 co-infected women from 34 weeks gestation to 12 months postpartum, as previously described [14]. The original study was powered to detect at least a 0.50 log_10_ copies/ml difference in plasma HIV-1 RNA levels. The study was approved by Kenyatta National Hospital Ethical Review Committee and the University of Washington Institutional Review Board, and registered at ClinicalTrials.gov under Identifier NCT 00530777. All participants provided written informed consent.

### Participants

Women presenting to public clinics in Nairobi, Kenya for antenatal care between April 2008 and June 2009 were recruited if they resided in Nairobi and were HIV-1-seropositive by rapid tests according to local protocols. Pregnant women with HIV-1 who were ≥18 years of age had HIV-1 confirmed by enzyme-linked immunosorbant assay (ELISA), HSV-2 antibodies, and CD4 count. HIV-1/HSV-2-seropositive women with CD4 counts >250 cells/µl and World Health Organization (WHO) clinical stage 1–2 were eligible.

Study participants received routine antenatal care and prevention of mother-to-child HIV-1 transmission (PMTCT) according to Kenya national guidelines: oral zidovudine (ZDV) 300 mg twice daily from 28 weeks gestation, oral ZDV at onset of labor and every 3 hours until delivery, single-dose nevirapine 200 mg at onset of labor, and ZDV or ZDV/lamivudine “tail” for 7 days postpartum. Baseline CD4 counts were measured prior to initiation of PMTCT regimens.

Enrollment took place at 34 weeks to allow completion of laboratory tests to determine eligibility. All women in the study received the following free of charge: antenatal and delivery care, co-trimoxazole prophylaxis, multivitamins in pregnancy, family planning, and prescribed outpatient medications.

### Study Procedures

WHO stage was determined at enrollment. Women were seen at 38 weeks gestation, within 48 hours of delivery, at 2, 6, 10, and 14 weeks postpartum, and at 6, 9, and 12 months postpartum. Antenatal study visits focused on obstetric care; postpartum visits included a physical exam and assessment of WHO stage. If women became eligible for antiretroviral therapy (ART) after enrollment, they were referred for treatment but continued to participate in the study. Counseling to promote adherence to study drug was done regularly. Women returned all unused pills to the study each month and at study termination. Adherence was measured by self-report of number of pills missed and by pill count at follow-up visits.

### Laboratory Methods

HIV-1 serostatus was confirmed by ELISA (Vironostika HIV Uni-Form II, bioMérieux, France) and HSV-2 serostatus was determined using ELISA (HerpeSelect, Focus Technologies, Cypress, CA) with a cutoff optical density (OD) ≥3.5 [15]. Tests were repeated if OD was between 1.1 and 3.4; women were eligible if the repeat assay was positive. CD4 counts were performed in Nairobi using flow cytometry (FACSCalibur or FACSCount, BectonDickinson, Franklin Lakes, NJ). HIV-1 RNA assays were conducted using a transcription-mediated amplification method validated for HIV-1 subtypes prevalent in Kenya (Gen-Probe Inc, San Diego, CA) [16]. HIV-1 RNA levels below the lower limit of detection (150 copies/ml) were recoded as half the value of the lower limit of detection.

### Study Outcomes

The primary outcomes of this nested study were CD4 counts and log_10_ plasma HIV-1 RNA levels compared between participants in the two study arms. Secondary outcomes included maternal morbidity, initiation of ART, mortality events and changes in WHO stage. Other study results, including effects of valacyclovir on breast milk HIV-1, genital HIV-1, and genital HSV-2 have been reported elsewhere [14]. A data safety monitoring board regularly evaluated adverse events; there were no criteria for stopping the study early other than safety.

### Statistical Methods

Analyses were conducted using Stata Version 10.0 (College Station, TX). All participants with a postpartum visit were included in this intention-to-treat analysis. Adequacy of randomization was assessed by chi-square and Fisher’s exact tests for categorical variables and Wilcoxon rank-sum tests for continuous variables comparing baseline values in both arms. Primary endpoints of CD4 counts and log_10_ HIV-1 plasma RNA levels at 6 months and 12 months were assessed using 2-sided t-tests and multivariate linear regression, controlling for baseline values. Cox proportional hazards regression was used to compare progression events in both arms. Adherence to study drug was measured by monthly pill counts and monthly self-report. Pill counts were totaled over the duration of the study and adherence was calculated as follows: (total pills dispensed – total pills missed)/total pills dispensed.

## Results

### Study Population

Women in both study arms had comparable baseline health status (Table 1). Most women lived in poverty in the urban slum neighborhoods surrounding the clinic. At enrollment, 85% of women in the placebo arm and 93% of women in the valacyclovir arm were WHO stage 1. Tuberculosis treatment was reported by 4% of women and 6% had prior herpes zoster. Participants in both study arms had similar baseline median CD4 counts and log_10_ HIV-1 plasma RNA levels. Almost all women (93% placebo, 100% valacyclovir) had started ZDV for PMTCT before study enrollment.

**Table 1:** Baseline Maternal Health Characteristics, by Treatment Arm.

Baseline Health Characteristic	Placebo n = 73	Valacyclovir n = 73	
	Median (IQR)or n (%)	Median (IQR)or n (%)	P value*
Age (years)	25 (22–29)	25 (22–30)	0.82
Prior STI	18 (25%)	20 (27%)	0.71
Prior syphilis	4 (5%)	4 (5%)	1.00
Prior GUD	13 (18%)	10 (13%)	0.5
Prior TB	3 (4%)	3 (4%)	1.00
Prior herpes zoster	5 (7%)	4 (6%)	0.73
On ARVs for PMTCT	68 (93%)	73 (100%)	0.02
WHO Stage 1	62 (85%)	68 (93%)	0.11
CD4 count (cells/µL)	481 (340–598)	452 (351–560)	0.78
Log_10_ HIV-1 plasma RNA level (copies/ml)	3.84 (3.43–.45)	3.99 (3.30–4.41)	0.86
No symptoms this pregnancy	44 (60%)	47 (64%)	0.61
Symptoms this pregnancy			
Fever	16 (22%)	18 (25%)	0.70
Malaria	12 (16%)	12 (16%)	1.00
Diarrhea	11 (15%)	15 (21%)	0.39
Cough	22 (30%)	27 (37%)	0.38
Weight loss	3 (4%)	9 (12%)	0.13
Itchy rash	4 (5%)	4 (5%)	1.00
Thrush	4 (5%)	4 (5%)	1.00

IQR  =  Interquartile range. USD  =  US Dollars. STI  =  sexually transmitted infection. GUD  =  genital ulcer disease. ARVs  =  antiretrovirals. TB  =  tuberculosis. PMTCT  =  prevention of mother-to-child transmission of HIV-1. *P value calculated using the Wilcoxon rank-sum test for continuous variables and chi-squared and Fisher’s exact test for categorical variables.

### Retention and Adherence

Retention was high: 146 (99%) participants had at least one postpartum visit, and 136 women (92%) were followed for 12 months. Adherence to study drug was high among study participants, and was sustained over 12 months postpartum. By pill count, overall adherence averaged 85% (84% placebo, 85% valacyclovir).

### CD4 Counts

**Figure 1:**
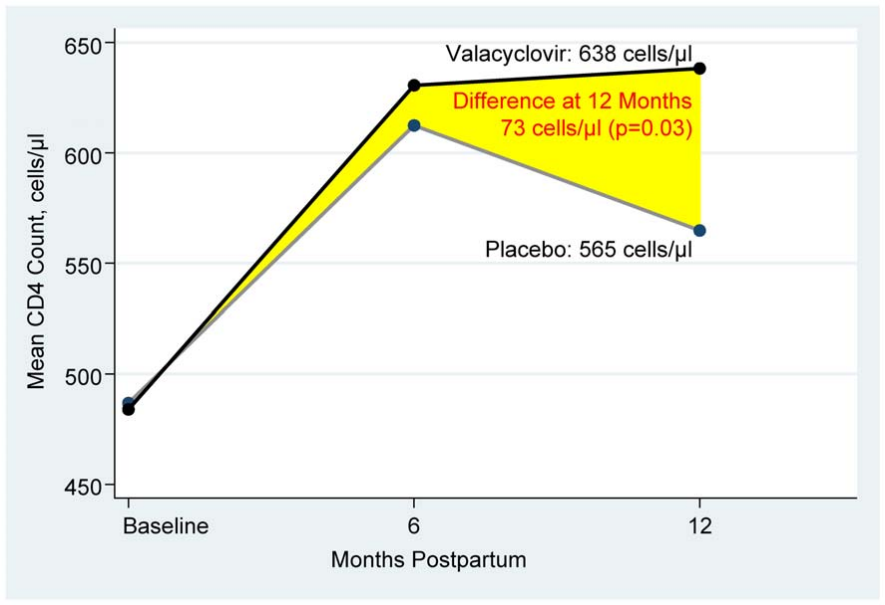
Change in CD4 count over time in participants on valacyclovir compared to placebo. Mean CD4 counts were compared at each timepoint by multivariate linear regression, adjusting for baseline values. Baseline CD4 counts were measured during pregnancy, prior to initiation of PMTCT antiretroviral short-course therapy.

Mean CD4 counts improved from baseline in both randomization arms (Table 2). Of the 116 participants with CD4 count results available at 6 months postpartum, mean CD4 increased from 484 cells/µl to 631 cells/µl in the valacyclovir arm and 487 cells/µl to 612 cells/µl in the placebo arm (*P* = 0.72). At 12 months, with 136 (93%) participants measured, we observed a higher mean CD4 in the valacyclovir arm than in the placebo arm (638 cells/µl vs. 565 cells/µl, respectively; *P* = 0.09). After adjusting for baseline CD4, the 12 month mean increase in CD4 counts was 73 cells/µl higher in the valacyclovir arm (*P*  = 0.03). Mean increase in CD4 count was 154 cells/µl in the valacyclovir arm, almost double the increase of 78 cells/µl in the placebo arm (Figure 1).

**Table 2:** Effect of Valacyclovir on Mean CD4 Count and Log_10_ Plasma HIV-1 RNA Level.

		Placebo	Valacyclovir	Difference, adjusted*		
	n	Mean (SD)	Mean (SD)	Mean	95% CI	*P*
**CD4 count (cells/µl)**						
Enrollment	146	487 (174)	484 (179)	−3	−61, 55	0.92
6 months postpartum	116	612 (232)	631 (309)	17	−58, 93	0.72
12 months postpartum	136	565 (206)	638 (292)	73	6, 140	0.03
**Log_10_ plasma HIV-1 RNA (copies/ml)**						
Enrollment	146	3.87 (0.83)	3.89 (0.95)	−0.01	−0.18, 0.17	0.95
6 months postpartum	136	4.40 (0.81)	3.98 (1.05)	−0.42	−0.65, −0.2	<0.001
12 months postpartum	136	4.53 (0.81)	4.10 (1.07)	−0.40	−0.63, −0.17	0.001

SD  =  standard deviation.

* method used: Multivariate linear regression models control for baseline plasma HIV-1 RNA levels for plasma HIV-1 RNA models, and baseline CD4 for CD4 models.

### HIV-1 RNA Levels

Mean plasma HIV-1 RNA levels were lowest in pregnancy (valacyclovir arm: 3.89 log_10_ copies/ml, placebo arm: 3.87 log_10_ copies/ml), when women were taking ZDV for PMTCT. Previously published data from this trial modeled plasma HIV-1 RNA from 6 weeks to 12 months postpartum and described an average 0.51 log copies/ml reduction in HIV-1 plasma RNA levels between study arms during that period [14]. In the present analysis, we report all antenatal, 6 month and 12 month plasma HIV-1 RNA data. During the postpartum period, HIV-1 RNA levels rebounded in both study arms after completion of maternal antiretrovirals (ARVs), but increases in HIV-1 RNA levels were smaller in the valacyclovir arm. At 6 months postpartum, after adjusting for baseline levels, plasma HIV-1 RNA levels were 0.42 log_10_ copies/ml lower in the valacyclovir arm than in the placebo arm (Table 2). At 12 months postpartum, adjusted plasma HIV-1 RNA levels were 0.40 log_10_ copies/ml lower in the valacyclovir arm than in the placebo arm; mean HIV-1 RNA increased by 0.21 log_10_ copies/ml in the valacyclovir arm, and increased by three times more, 0.66 log_10_ copies/ml, in the placebo arm (*P* = 0.001).

### Maternal Morbidity, Mortality, and Obstetric Outcomes

Despite provision of free care to study participants, there were multiple poor maternal outcomes. Overall, 3 women (2%) died during the study (2 placebo, 1 valacyclovir) and 7 (5%) were hospitalized (5 placebo, 2 valacyclovir). Poor birth outcome with fetal demise occurred in 3 women (2 placebo, 1 valacyclovir) who had third-trimester stillbirths. Other morbidities included tuberculosis (6 women), diarrhea/colitis (26 women), malaria (20 women) and pneumonia (7 women). Overall, 6 women progressed to WHO Stage 3 or 4 during the study, 3 in each arm. Sixteen women (7 on placebo, 9 on valacyclovir) had a progression event (death, CD4<250 cells/µl, WHO stage 3–4; or opportunistic infection). Cox proportional hazards regression models assessing time to disease progression events revealed no significant differences in risk of any progression events, but the study was not powered for these outcomes and events were few. There were no serious adverse events related to use of valacyclovir.

## Discussion

Our study of daily valacyclovir during late pregnancy and postpartum showed a significant increase in CD4 counts among women in the valacyclovir arm at 12 months postpartum. CD4 is the most reliable marker of HIV-1 disease progression and morbidity in pregnant and postpartum African women [17]. Although all women in this study received short-course maternal ARVs for PMTCT, the rise in maternal CD4 count in the valacyclovir arm was sustained months after maternal ARVs for PMTCT had been discontinued. This improvement in CD4 is plausible since we concurrently observed less increase in plasma HIV-1 RNA among women in the valacyclovir arm, modeled over the entire study period. Given that asymptomatic African patients with HIV-1 lose an estimated 30–60 CD4 cells/µl per year [18], [19], [20], improved CD4 counts of the magnitude observed in this study could translate into improved outcomes and delayed need for ART for postpartum women.

This is the first randomized trial reporting results of valacyclovir on HIV-1 disease progression specifically among pregnant women in Africa. Other studies in African non-pregnant HIV-1 infected populations have noted benefits of acyclovir, including the landmark trial by Lingappa *et al*, noting a 16% reduction in HIV-1 disease progression with acyclovir therapy [12], and a randomized controlled trial in Uganda of daily acyclovir among HIV-1/HSV-2 seropositive adults with CD4 counts 300–400 cells/µl that showed a 25% reduction in disease progression after 24 months [13]. Mugwanya *et al* published data showing that higher doses of valacyclovir may have more potential to impact HIV-1 disease [21]. Modeling studies have predicted similar declines in HIV-1 disease progression with herpes suppressive therapy [22], [23]. A recent cost-effectiveness analysis indicates that HSV-2 suppression to delay ART could be cost-effective in South Africa; HSV-2 suppression could potentially be more cost-effective in other African countries where labor costs are lower [24]. Further, long-term studies of acyclovir in HIV-1/HSV-2 co-infected adults showed no evidence of significant HIV-1 resistance mutations [25], and no evolution of HSV-2 resistance to acyclovir [26], even when adherence was sub-optimal, indicating that prolonged use of anti-herpes medication has few downsides.

Consistent with prior studies of daily acyclovir and valacyclovir, we found high adherence (86%) during the peri- and postpartum periods. Women successfully adhered to twice daily valacyclovir despite extreme poverty, motherhood, and even when concealing both HIV status and study participation. We believe that community health workers were instrumental in supporting adherence in our study, and conclude that widespread use of this therapy should be possible in most African settings, especially if accompanied by support for adherence.

The randomized design of this study proved helpful in comparing CD4 counts in pregnant and postpartum women, which fluctuate due to hemodilution of pregnancy and use of ARVs for PMTCT. Other strengths of this study include its extended duration of follow-up, excellent retention, and implementation within existing PMTCT programs. The small number of women in our study and the relatively brief follow-up time limited our ability to comment on disease progression events. Although valacyclovir is now a generic medication, it is not widely available in resource-limited settings, and continues to be relatively expensive.

What would be the role of valacyclovir, given the current push for elimination of PMTCT? Efforts to scale-up HIV treatment include increasing recognition of the benefits, both reduced infant HIV transmission and improved maternal outcomes, when pregnant women are started on ART or prophylaxis as early as possible [27], especially mothers who need ART for their own health. WHO guidelines recommend that, in addition to providing ART to all pregnant women with CD4≤350 cells/µl, countries choose either short-course maternal ZDV during pregnancy and infant prophylaxis during breastfeeding (“Option A”) or maternal ART during pregnancy and breastfeeding (“Option B”) as preferred interventions to eliminate MTCT [28]. Some countries, such as Malawi, are adopting “Option B-Plus” and placing women on lifelong ART [29], but there are ongoing concerns about retaining women in care who initiate ART solely for PMTCT [30], [31]. Thousands of women will continue to receive only ZDV prophylaxis under “Option A;” this trial shows that valacyclovir could benefit these women, especially since short-course regimens in pregnancy will continue to have a place in PMTCT programming for the foreseeable future.

In conclusion, pregnant and postpartum women randomized to 500 mg of valacyclovir twice daily had greater improvements in CD4 counts and lower postpartum maternal HIV-1 RNA levels when compared to women randomized to placebo. HIV-1 infected women and their children in Africa continue to face serious health consequences and excess mortality due to a lack of comprehensive and timely HIV-1 care and prevention. There is an urgent need to improve maternal CD4 counts and reduce maternal HIV-1 disease progression to promote maternal and child survival. The promising CD4 data from this trial indicate that valacyclovir may be a tool to reduce progression events in women. Further studies are needed to determine whether peripartum valacyclovir therapy may be useful to improve maternal outcomes in settings where access to ART is limited and HSV-2 co-infection is prevalent.
